# Precise measurement of ultra-narrow laser linewidths using the strong coherent envelope

**DOI:** 10.1038/srep41988

**Published:** 2017-02-09

**Authors:** Shihong Huang, Tao Zhu, Min Liu, Wei Huang

**Affiliations:** 1 Key Laboratory of Optoelectronic Technology & Systems (Ministry of Education), Chongqing University, Chongqing 400044, China

## Abstract

Laser linewidth narrowing down to kHz or even Hz is an important topic in areas like clock synchronization technology, laser radars, quantum optics, and high-precision detection. Conventional decoherence measurement methods like delayed self-heterodyne/homodyne interferometry cannot measure such narrow linewidths accurately. This is because a broadening of the Gaussian spectrum, which hides the laser’s intrinsic Lorentzian linewidth, cannot be avoided. Here, we introduce a new method using the strong coherent envelope to characterize the laser’s intrinsic linewidth through self-coherent detection. This method can eliminate the effect of the broadened Gaussian spectrum induced by the 1/*f* frequency noise. We analyze, in detail, the relationship between intrinsic laser linewidth, contrast difference with the second peak and the second trough (CDSPST) of the strong coherent envelope, and the length of the delaying fiber. The correct length for the delaying fiber can be chosen by combining the estimated laser linewidth (Δ*f*_est_) with a specific CDSPST (ΔS) to obtain the accurate laser linewidth (Δ*f*). Our results indicate that this method can be used as an accurate detection tool for measurements of narrow or super-narrow linewidths.

Because lasers with narrow linewidths have long coherence lengths, they are widely used for high precision and ultra-long distance detection in e.g. optical atomic clocks^1^,^2^, laser radars^3^,^4^, and distributed sensing^5^. The performance of these optically coherent systems i.e. their range, precision, sensitivity, and noise strongly depend on the span of the laser’s Lorentzian linewidth^6^,^7^,^8^,^9^,^10^. Hence, the accurate determination of the Lorentzian linewidth of the narrow laser is very important.

The conventional methods for laser linewidth measurements include delayed self-heterodyne/homodyne interferometers (DSHIs)^11^,^12^ or extended variations, e. g. loss-compensated recirculating DSHIs^13^,^14^,^15^, and polarization-insensitive Michelson DSHIs^16^. There are two components included in the line shape of the detected power spectrum using the above methods. One is the natural linewidth (Lorentzian linewidth) that results from white frequency noise, and the other is the approximate Gaussian linewidth that results from the 1/*f* frequency noise and the long delaying fiber^9^,^17^. Though Voigt fitting has been used to separate the Lorentzian spectrum from the approximate Gaussian spectrum, the ratio of the two components is hard to be determined. In addition, the Lorentzian component would be masked by the Gaussian component arising from the 1/*f* noise and the ultra-long fiber used for ultra-narrow linewidth detection^17^,^18^. Also, direct heterodyne beat between two almost identical laser sources can directly reflect the linewidth of the laser sources due to the incoherence of these two lasers without considering the effect of 1/*f* frequency noise^19^,^20^, but it is difficult to find another ultra-stable laser source with super-narrow linewidth and almost the same center wavelength as reference. Moreover, if the detected laser has arbitrary wavelength, it is more difficult to find an ultra-stable laser with arbitrary wavelength.

In order to obtain the accurate value for the Lorentzian linewidth, the effect of the broadening Gaussian linewidth induced by 1/*f* noise needs to be eliminated. L. B. Mercer, H. Ludvigsen, L. E. Richter and S. H. Huang^9^,^12^,^21^,^22^,^23^ demonstrated that short delay self-heterodyne interferometry (SDSHI) is a good method to filter out 1/*f* noise. However, it is still not clear how long the delaying fiber should be to eliminate the effect of 1/*f* noise to obtain the accurate Lorentzian linewidth for different levels of the laser linewidth.

We have previously proposed an accurate method to obtain the Lorentzian linewidth by comparing the contrast difference between the second peak and the second trough (CDSPST) of the coherent envelope of the power spectrum using SDSHI^22^. In this letter, we further investigate the relationship between the laser linewidth (Δ*f*), the CDSPST value of the coherent envelope (ΔS), and the length of the delaying fiber (L). Moreover, we modify the model of CDSPST value to obtain a more accurate linewidth. It is verified, both theoretically and experimentally, that the Gaussian spectrum induced by the 1/*f* noise can be eliminated using this method. A suitable length for the delaying fiber (L) can be chosen by combining the estimated laser linewidth (Δ*f*_est_) and a certain level of CDSPST (ΔS) to obtain its accurate laser linewidth (Δ*f*). This can also provide a detailed guideline to obtain the accurate Lorentzian linewidth for laser linewidths of different magnitudes.

## Module Analysis

From the study of ref. 22, the CDSPST (ΔS) value was chosen to reflect the Lorentzian laser linewidth because the CDSPST (ΔS) value near the center frequency has the advantages of a small detection error and higher stability compared to the center frequency. Considering the output-power spectrum S(*f*, Δ*f*) = S_1_S_2_ of the DSHI (see Supplementary Figure S1), where S_1_ is the Lorentzian spectrum and S_2_ is the periodic modulation power spectrum, the value of CDSPST (ΔS) can be expressed as


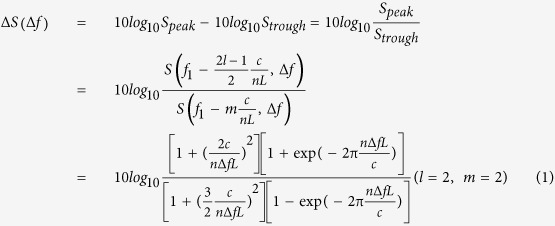


where *f* is the measurement frequency, *f*_1_ is the AOM frequency shift, *n* is the fiber optic refractive index, *c* is the light speed, *L* is the length of the delaying fiber, and Δ*f* is the full-width-half-maximum (FWHM) of the power spectrum (Lorentzian linewidth). The parameters *l* = 2, 3, 4…… and *m* = 1, 2, 3……represent the positions of different peaks and troughs of the coherent envelope, respectively. The value of CDSPST (ΔS) is achieved when the parameters *l* and *m* are set to 2 and 2, respectively.

**Figure 1:**
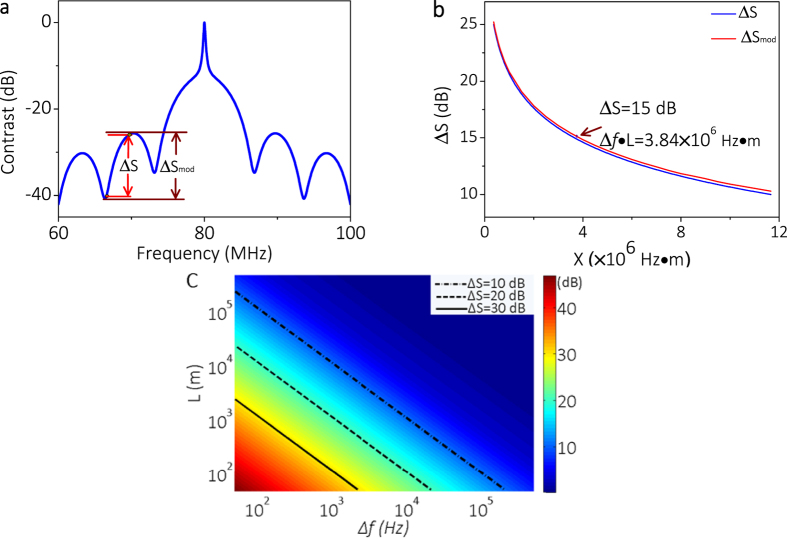
(**a**) Simulated normalized power spectrum with Δ*f* = 125 kHz and *L* = 30 m using S(*f*, Δ*f*) = S_1_S_2_, (**b**) different ΔS values for different X = Δ*f·L* using the simplified equation (1) (blue curve) and the real values of CDSPST (ΔS_mod_) for different X = Δ*f·L* from its simulated power spectrum using S(*f*, Δ*f*) = S_1_S_2_(red line), and (**c**) The relationship between the laser linewidth (Δ*f*), the value for CDSPST of the coherent envelope (ΔS), and the length of the delaying fiber (*L*) using S(*f*, Δ*f*) = S_1_S_2_.

Figure 1(a) shows the simulated normalized power spectrum S with a 125 kHz laser linewidth (Δ*f* = 125 kHz) and a 30 m delaying fiber (*L* = 30 m) using S(*f*, Δ*f*) = S_1_S_2_. Because the output power spectrum S is the product of the Lorentzian spectrum S_1_ and the periodic modulation power spectrum S_2_, the periodicity of the coherent envelope is determined by the periodic modulation power spectrum S_2_ (see Supplementary Figure S1). The actual positions of the second peak and trough would move slightly compared with *f*_1_−3*c*/(2*nL*) and *f*_1_−2*c*/(*nL*) (see Fig. 1(a)). Here, ΔS = 14.89 dB and ΔS_mod_ = 15.11, where ΔS is achieved by equation (1), and ΔS_mod_ is the real CDSPST value from its simulated power spectrum using S(*f*, Δ*f*) = S_1_S_2_. From the simplified equation (1) and the simulation results (blue curve) (see Fig. 1(b)), we find that the value X = Δ*f*·*L* is a constant when the value of CDSPST (ΔS) is fixed. Comparing the simulated results (blue curve) from the simplified equation (1) with the real CDSPST value (ΔS_mod_) (red line) from its simulated power spectrum by S(*f*, Δ*f*) = S_1_S_2_, the value of CDSPST (ΔS) should be modified as


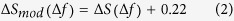


The CDSPST (ΔS) value mentioned below is ΔS_mod_. For an estimated laser linewidth (Δ*f*_est_) which is provided by the manufacturer, the length of the delaying fiber should be chosen as *L*≅3.845 × 10^6^/Δ*f*_est_ if the value of CDSPST (ΔS) is chosen to be ~15 dB from the coherent envelope of the power spectrum. The accurate detection linewidth (Δ*f*) is determined by combining the experimentally detected CDSPST (ΔS) value and the chosen delaying fiber length L(see Fig. 1(c)).

## Results and Discussion

The experimental setup and its principle has been described in Method and Supplementary (see Supplementary 1), respectively. Laser source whose linewidth ~150 kHz provided by the manufacturer is used to obtain the accurate Lorentzian linewidth.

**Figure 2:**
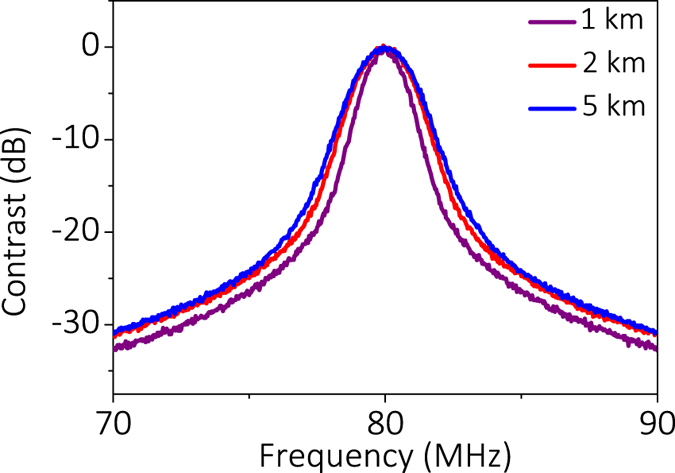
Detected normalized power spectrum for different delaying fibers utilizing the DSHI method.

Figure 2 shows the detected power spectrum for different delaying fibers utilizing the laser with ~150 kHz linewidth with the DSHI method. The detected linewidth is 1.000 MHz with a 1 km delaying fiber, 1.288 MHz with a 2 km delaying fiber, and 1.423 MHz with 5 km delaying fiber respectively if we use 3-dB linewidth directly (see Table 1). The detected 3-dB linewidth is much larger than that given by the manufacturer because of the broadening Gaussian linewidth induced by the 1/*f* noise and the delaying fiber^9^,^24^,^25^,^26^,^27^,^28^,^29^,^30^. The detected linewidth is 225 kHz with the 1 km delaying fiber, 320 kHz with the 2 km delaying fiber, and 352 kHz with the 5 km delaying fiber, respectively, if we use 20-dB linewidth fitting. The broadening effect by the Gaussian linewidth induced by the 1/*f* in the center frequency is decreased when we use the 20-dB linewidth fitting. The linewidth broadening phenomenon using 3-dB or 20-dB linewidth fitting is consistent with equation S2 (see Supplementary 2), which indicates that the longer the delaying fiber, the larger the Gaussian linewidth is for the center frequency.

**Figure 3:**
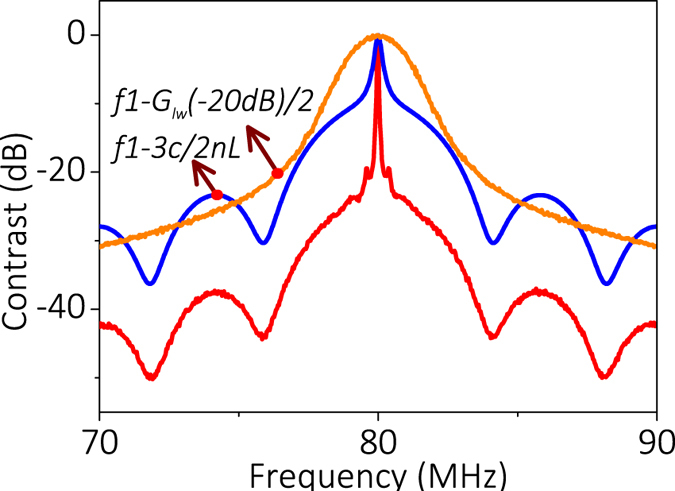
Detected normalized power spectrum with 5000 m (orange line) and 50 m (red line) delaying fiber, respectively, (the output power is 5 mW) and the simulated normalized power spectrum with 50 m delaying fiber and 125 kHz linewidth (blue line).

Because the estimated laser linewidth (Δ*f*_est_) provided by the manufacturer is ~150 kHz, 5 km delaying length is long enough to eliminate the coherency between two beams of the Mach-Zehnder interferometer (MZI) structure using traditional DSHI. The 20-dB linewidth of the detected power spectrum is ~7.04 MHz (see Fig. 3). Its FWHM is ~7.04 MHz/20 = 352 kHz, when using the traditional 20-dB linewidth fitting. Considering that the maximum Gaussian linewidth must be narrower than the detected linewidth using traditional DSHI methods, we can use the power spectrum detected by DSHI to determine the suitable length for the delaying fiber, which can eliminate the influence of the Gaussian spectrum induced by the 1/*f* noise. It can be ensured that the CDSPST (ΔS) value cannot be affected by the Gaussian linewidth when the position of the second peak *f*_1_-3*c*/(2*nL*) (using SDSHI) is far away from the position *f*_1_-G_*lw*_ (−20 dB)/2 (using DSHI), where G_*lw*_ (−20 dB) can be replaced with the detected power spectrum (D_*lw*_(−20 dB)) using the 5 km delaying fiber. The length of the delaying fiber can be determined with the following relationship:


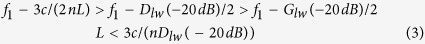


From this equation, we can see that as for *L* < 85.72 m (*D*_*lw*_(−20 dB) = 7.04 MHz) the effect of the Gaussian spectrum induced by the 1/*f* laser noise near the center frequency can be ignored. In the experiment, 50 m of delaying fiber was chosen to eliminate the effect of the Gaussian linewidth to obtain the accurate Lorentzian linewidth with our method. From the detected normalized power spectrum for 5 km and the simulated normalized power spectrum fór 50 m (see Fig. 3), we find that the position of the second peak *f*_1_−*3c/2nL* for the 50 m delaying fiber is far from position *f*_1_−*D*_*lw*_(−20 dB)/2. Hence, CDSPST (ΔS) cannot be affected by the Gaussian linewidth. The detected CDSPST (ΔS) for 50 m is 12.90 dB and its corresponding X = Δ*f·L* = 6.249 × 10^6^ Hz·m (see Fig. 1(b)). The accurate detected Lorentzian linewidth is Δ*f* = 6.249 × 10^6^/50 = 125 kHz.

**Figure 4:**
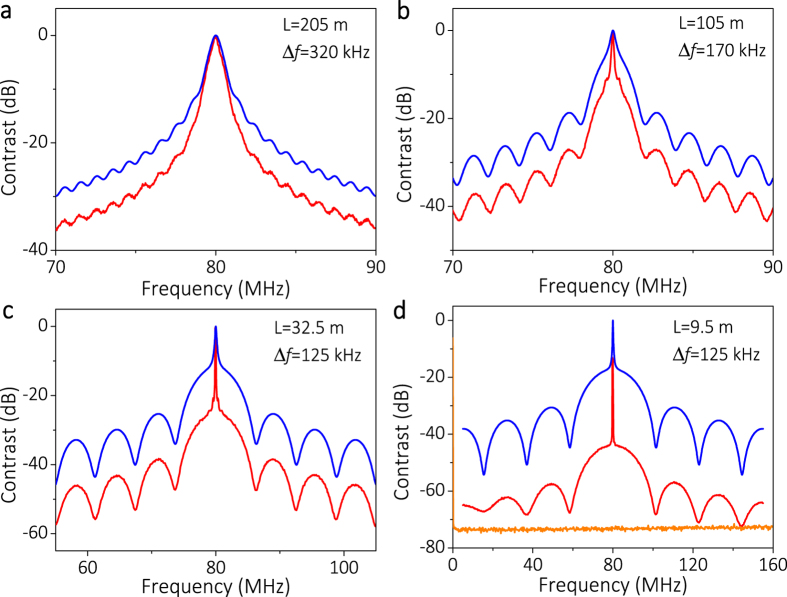
The detected normalized power spectrum (red line) and the simulated normalized power spectrum (blue line) with 5 mW output power. (**a**) *L* = 205 m, Δ*f* = 320 kHz, (**b**) *L* = 105 m, Δ*f* = 170 kHz, (**c**) *L* = 32.5 m, Δ*f* = 125 kHz, (**d**) *L* = 9.5 m, Δ*f* = 125 kHz; the horizontal line is the noise of the detected system.

Based on the detected CDSPST (ΔS) of 14.80 dB for 32.5 m (see Fig. 4(c)) and its corresponding X = Δ*f·L* = 4.035 × 10^6^ Hz·m (see Fig. 1(b)), the accurate Lorentzian linewidth can be obtained as Δ*f* = 4.035 × 10^6^/32 = 126 kHz. The accuracy of the method can be ensured by comparing the well-fitted curves between the simulated and the detected power spectra with 50 m delaying fiber (see Fig. 3) and the 32.5 m delaying fiber (see Fig. 4(c)).

As for the delaying fiber *L* > 85.72 m (see Fig. 4(a) and (b)), the detected linewidth is ~170 kHz for the 105 m delaying fiber and ~320 kHz for the 205 m delaying fiber. The detected broadening linewidth is mainly induced by the Gaussian line shape because the longer delaying fiber, the larger is the detected linewidth. Therefore the delaying fiber should be chosen according to: *L* < *3c*/(*n D*_*lw*_(−20 dB)).

When the delaying fiber (*L* = 9.5 m) is too short (see Fig. 4(d)), the detected power spectrum does not fit the simulated power spectrum. This is because the shorter the delaying fiber, the smaller are the values for the second peak and the second trough of the power spectrum. Once the second trough decreases to or below the noise floor of the spectrum analyzer, it will be masked by the noise of the spectrum analyzer, which will lead to an incorrect CDSPST (ΔS) value and the corresponding detected linewidth.

Because a long delaying fiber broadens the laser linewidth and a short delaying fiber leads to an incorrect coherent envelope, the length of the delaying fiber should be chosen properly. The CDSPST (ΔS) determined by the chosen length of the delaying fiber is used to reflect the laser linewidth. It should be chosen in the range between 10 dB and 30 dB (see Fig. 1(c)), because a low CDSPST (ΔS) decreases the detected accuracy and a high CDSPST (ΔS) is masked by the noise of the ESA. Table 2 shows different delaying fibers for different levels of Δ*f* with suitable CDSPST (ΔS) values when the coherent envelope is under good conditions.

From Table 2, the relationship between the estimated linewidth (Δ*f*_est_) and CDSPST (ΔS) can be set approximated using the following expression


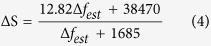


**Figure 5:**
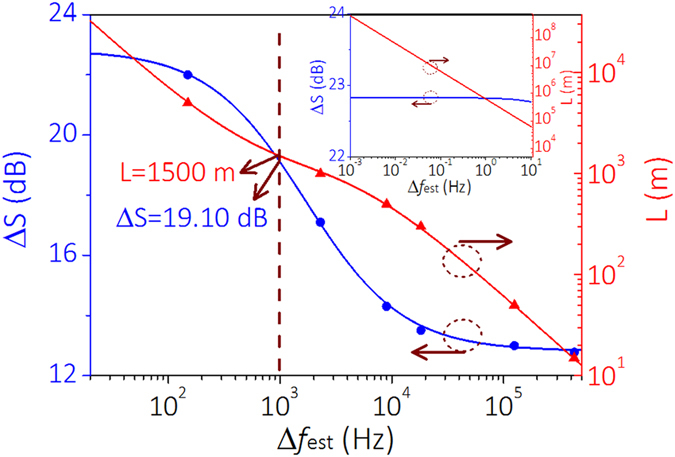
The suitable CDSPST (ΔS) value (blue line) for different levels of Δ*f*_est_ and its suitable corresponding lengths of delaying fiber to be chosen (red line).

The suitable length of the delaying fiber can be ensured by combing equation (1) and equation (4). Figure 5 shows the ΔS and the length of the delaying fiber that should be chosen for different laser linewidth detection. For instance, if we want to obtain the accurate Lorentzian linewidth ~Δ*f*_est_ = 1 kHz, CDSPST (ΔS) can be chosen as ~19.10 dB and its suitable corresponding delaying fiber length is ~1500 m. It can be seen that equation (4) and Fig. 5 give us a detailed guideline for how to obtain an accurate Lorentzian linewidth for different magnitudes of laser linewidth detection.

The small diagram in Fig. 5 shows the suitable CDSPST (ΔS) value (blue line) for the 0.001 Hz~10 Hz ultra-narrow linewidth Δ*f*_est_ and its suitable corresponding length for the delaying fiber (red line). This method is suitable for the detection of accurate ultra-narrow linewidths. However, although this method can detect laser linewidths very accurately, one limitation exists. Based on the small diagram in Fig. 5 we can infer that the ultra-long delaying fiber should also be used for super-narrow linewidth detection (~12000 km delaying fiber is used for ~50 mHz laser linewidth detection), which will lead to a small frequency range between the second peak and second trough, and a higher bandwidth resolution for the electric spectrum analyzer (ESA) should be used to detect the CDSPST (ΔS) value.

Although this method has its limitation, it uses a shorter delaying fiber and higher accuracy than the traditional method. Furthermore, a delaying fiber less than 10 km long is available for normal ultra-narrow linewidth (larger than Hz level) detection (see Fig. 5). In other words, this method has advantages for accurate ultra-narrow linewidth detection.

Actually, some references such as refs 12,22,23 have achieved the coherent envelope of the power spectra by using short delaying fiber, and we can use our method to modify their detected linewidth. For instance, the value of CDSPST (ΔS) is ~18 dB with ~2.27 km delaying fiber in ref. 23, then its corresponding linewidth is ~850 Hz with this method.

## Conclusions

In summary, we have investigated both theoretically and experimentally, the relationship between the Lorentzian laser linewidth (Δ*f*), the CDSPST (ΔS) value, and the length of the delaying fiber (L) using SDSHI. In order to obtain the accurate Lorentzian linewidth, the real CDSPST (ΔS) value was modified through the simulation of the power spectrum and the suitable length for the delaying fiber to eliminate the Gaussian linewidth induced by 1/*f* noise was identified. We found that a suitable length of the delaying fiber can be selected for different magnitudes of laser linewidth (Δ*f*_est_) based on SDSHI to obtain the accurate Lorentzian linewidth (Δ*f*). This method represents an accurate detection tool for narrow or ultra-narrow linewidth measurements, which is of great importance for the field of ultra-narrow linewidth laser study and its applications.

## Methods

### Measurement methods

**Figure 6:**
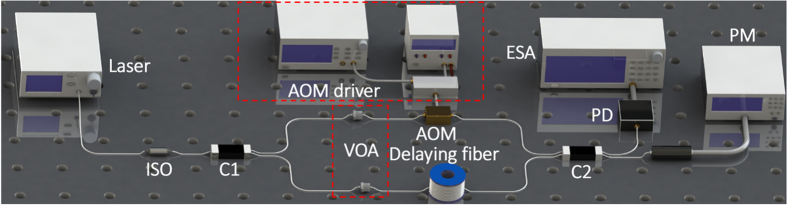
Schematic of the experimental setup. ISO: optical isolator, C1, C2: opto-couple (50/50), VOA: optical attenuator, AOM: acoustic optical modulator. ESA: electric spectrum analyzer, PD: photodetector, PM: power meter.

The direct detection of the laser linewidth using delayed self-heterodyne is traditionally carried out with a Mach-Zehnder interferometer (MZI)^11^,^12^,^21^,^22^ (see Fig. 6). The acoustic optical modulator (AOM: 80 MHz, Gooch & Housego) is used to generate a frequency shift. The combining laser is monitored by a photoelectric detector (PDB430C, 350 MHz, Thorlabs). The laser output is detected with an electric spectrum analyzer (FSV, 10Hz-30GHz, ROHDE&SCHWARZ). To ensure the detector is unsaturated and almost the same detected power for the two beams from the MZI is generated, two variable optical attenuators (VOA) were inserted into the MZI setup. The principle of this experimental setup has been described in Supplementary (see Supplementary 1).

## Additional Information

**How to cite this article**: Huang, S. *et al*. Precise measurement of ultra-narrow laser linewidths using the strong coherent envelope. *Sci. Rep.* **7**, 41988; doi: 10.1038/srep41988 (2017).

**Publisher's note:** Springer Nature remains neutral with regard to jurisdictional claims in published maps and institutional affiliations.

## Supplementary Material

Supplementary Information

## Figures and Tables

**Table 1:** Different detected linewidth for different fitting.

Delayed length	DSHI (−3 dB)	DSHI(−20 dB)
1 km	2 MHz/2 = 1.000 MHz	5.1 MHz/20 = 225 kHz
2 km	2.576 MHz/2 = 1.288 MHz	6.4 MHz/20 = 320 kHz
5 km	2.846 MHz/2 = 1.423 MHz	7.04 MHz/20 = 352 kHz

**Table 2:** Different lengths of delaying fibers for different levels of Δ*f* with suitable CDSPST (ΔS).

Δ*f*_est_ (kHz)	Delaying length (m)	ΔS (dB)	Δ*f* (kHz)
0.1	5000	21.98	0.15
2.5	1000	17.11	2.30
10	500	14.31	9.10
20	300	13.50	18.20
150	50	12.98	125
450	15	12.78	430
