# Supplementary material for: Precise measurement of ultra-narrow laser linewidths using the strong coherent envelope
Source: Sci Rep. 2017 Feb 9;7:41988. doi: 10.1038/srep41988 (PMC5299402; doi:10.1038/srep41988)
Supplement: Supplementary Information [file srep41988-s1.pdf]

SUPPLEMENTARY INFORMATION

Precise measurement of ultra-narrow laser linewidths using the strong coherent envelope

Shihong Huang, Tao Zhu, Min Liu, and Wei Huang

Key Laboratory of Optoelectronic Technology & Systems (Ministry of Education), Chongqing University, Chongqing 400044, China.

1. Numerical simulation module details based on SDSHI

The detected power spectrum density can be summarized as the following equation from [1-5].

$$S(f,\Delta f) = S_1 S_2 + S_3 \qquad \text{S1}$$

Here

$$S_1 = \frac{P_0^2}{4\pi}\frac{\Delta f}{\Delta f^2 + (f - f_1)^2} \qquad \text{S1 (a)}$$

$$S_2 = 1 - \exp(-2\pi\Delta f\tau_d)\left[\cos[2\pi(f \pm f_1)\tau_d] + \Delta f\frac{sin[2\pi(f \pm f_1)\tau_d]}{f \pm f_1}\right] \qquad \text{S1 (b)}$$

$$S_3 = \frac{\pi P_0^2}{2}\exp(-2\pi\Delta f\tau_d)\,\delta(f \pm f_1) \qquad \text{S1 (c)}$$

where P_0 is the detected optical power by PD, f is the measurement frequency, f_1 is the AOM frequency shift, τ_d (τ_d=L/c, L is the length of the delaying fiber, c is the speed of light) is the time delay of one path with respect to the other path, and Δf is the full-width-half-maximum (FWHM) of the power spectrum (Lorentzian linewidth). As for EqS1(c), when $f \neq f_1$, $\delta(f \pm f_1)=0$, $S_3 = 0$ and $f = f_1$, S_3=infinite, the power spectrum S can be simplified to be $S(f, \Delta f)=S_1S_2$ whereas the detected power spectrum is unstable at $f = f_1$. All simulated normalized power spectra were computed using $S(f, \Delta f)=S_1S_2$.

From Eq. S1, we can see that the power spectrum S is the product of the Lorentzian spectrum S_1 and the periodic modulation power spectrum S_2. Figure S1 shows the simulated normalized power spectrum for S (brown line), S_1 (red line), and S_2 (blue line), respectively, with a 1 kHz laser linewidth (Δf=1 kHz) and 1500 m of delaying fiber (L=1500 m). If 500 km delaying fiber is used to detect the 1 kHz laser linewidth, the amplitude for S_2 is so small that the power spectrum S is almost equal to Lorentzian spectrum S_1, and this is the classical DSHI used to detect the laser linewidth. However, for

the actual experiment, the use of such a long delaying fiber would induce a large Gaussian spectrum by $1/f$ noise for the center frequency [5-14], which could mask the Lorentzian spectrum. This is why it is difficult to detect accurately the narrow linewidth using traditional DSHI. If τ_d is much smaller than the laser coherent time $\tau_c=1/(2\pi\Delta f)$, the amplitude of S_2 is too large to be neglected and it will be periodically superimposed on the Lorentzian line shape S_1 to finally form S shown in figure S1.

Since the power spectrum S is the product of the Lorentzian spectrum S_1 with the periodic modulation power spectrum S_2, the Lorentzian linewidth Δf can also be reflected by its coherent envelope. This method can also eliminate the effect of the Gaussian spectrum for the center frequency. From this point of view, our method provides a new way to accurately detect laser linewidths [5].

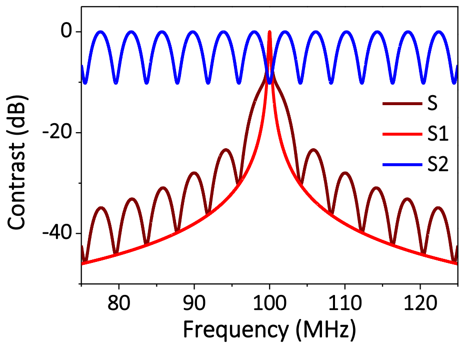

Figure S1 | The simulated normalized power spectra for S (brown line), S_1 (red line), S_2 (blue line), respectively, with Δf=1 kHz and L=1500 m.

2. Influence of the Gaussian linewidth

In reference [15], the Gaussian linewidth induced by the $1/f$ noise is about

$$G_{lw} = \frac{1}{\pi}\sqrt{\frac{2k\ln 2}{\pi}\left(4.3+\ln\frac{4.3k\tau_d^{2.1}}{\pi}\right)}\quad Hz\ FWHM \qquad \text{S2}$$

where k depends on the type of phase-locked loop and the acceptable phase-error variance [15-22], and $\tau_d=nL/c$. If we want to reduce the Gaussian linewidth induced by the $1/f$ noise to obtain the Lorentzian linewidth, the level k should be decreased and the delaying fiber L should be shortened. Since the level for k is difficult to detect and different lasers have different k values, the value for G_{lw} is difficult to be determined. It has been shown that shortening the length of the delaying fiber is a valuable way to eliminate Gaussian linewidth. Since the power spectrum detected by DSHI is the convolution of the Lorentzian spectrum and the approximately Gaussian spectrum [15, 23, 24], the linewidth of the detected power spectrum must be larger than the linewidth of the Gaussian spectrum. Therefore, in this letter,

we use the power spectrum detected using DSHI to displace the Gaussian spectrum and determine the suitable length for the delaying fiber to eliminate the effect of the Gaussian spectrum induced by the $1/f$ noise.

References

1. Okoshi, T., Kikuchi, K., Nakayama, A. Novel method for high-resolution measurement of laser output spectrum. *Electron. Lett.* **16**, 630–631 (1980).
2. Richter, L. E., Mandelberg, H. I., Kruger M. S. & McGrath, M. S., Linewidth determination from self-heterodyne measurements with sub-coherence delay times. *IEEE J. Quantum. Electron*. **22**, 2070-2074 (1986).
3. Ludvigsen, H., Tossavainen, M., Kaivola, M., Laser linewidth measurements using self-homodyne detection with short delay. *Optics Commu.* **155**, 180-186 (1998).
4. Canagasabey, A., Michie, A., Canning, J., Holdsworth, J. Fleming, S., Wang H. C., & Åslund, M. L. A comparison of delayed self-Heterodyne interference measurement of laser linewidth using Mach-Zehnder and Michelson interferometers. *Sensor*. **11**, 9233-9241 (2011).
5. Huang, S. H., Zhu, T., Cao, Z. Z., Liu, M., Deng, M., Liu, J. G. & Li, X. Laser Linewidth Measurement Based on Amplitude Difference Comparison of Coherent Envelope, *IEEE Photon. Technol. Lett*. **28**, 759-762 (2016).
6. Mercer, L. B. 1/f frequency noise effects on self-heterodyne linewidth measurements. *J. Lightwave Technol.* **9**, 485–493 (1991).
7. Kikuchi, K. Effect of l / f type FM noise on semiconductor laser linewidth residual in high power limit. *IEEE J. Quantum Electron*. QE-**25**, 684-688 (1989).
8. Chen, M., Meng, Z., Wang, J. & Chen, W., Ultra-narrow linewidth measurement based on Voigt profile fitting. *Opt. Express*. **23**, 6803-6808 (2015).
9. Bruce, S. D., Higinbotham, J., Marshall, I. & Beswick, P. H., An analytical derivation of a popular approximation of the Voigt function for quantification of NMR spectra. *J. Magn. Reson*. **142**, 57-63 (2000).
10. NAZARATHY, M., SORIN, W. V. BANEY, D. M. & Newton, S. A. Spectral Analysis of Optical Mixing Measurements. *J. Lightwave Technol.* **7**, 1083-1096 (1989).
11. Kikuchi, K., Impact of 1/f-type FM noise on coherent optical communications. *Electron. Lett.* **23**, 885–887 (1987).
12. Fleming, M. W. & Mooradian, A. Spectral characteristics of external cavity controlled semiconductor lasers. *IEEE J. Quantum. Electron.* **17**, 44-59 (1981).
13. Yamamoto, Y., Mukai, T. & Saito, S. Quantum phase noise and linewidth of a semiconductor laser. *Electron. Lett.* **17**, 327-329 (1981).
14. Daino, B., Spano, P., Tamburrini, M. & Piazzolla, S. Phase noise and spectral line shape in semiconductor lasers. *IEEE J. Quantum. Electron*. **19**, 630-631 (1983).
15. Mercer, L. B. 1/f frequency noise effects on self-heterodyne linewidth measurements. *J. Lightwave Technol.* **9**, 485–493 (1991).

16. NAZARATHY, M., SORIN, W. V. BANEY, D. M. & Newton, S. A. Spectral analysis of optical mixing measurements. *J. Lightwave Technol.* **7**, 1083-1096 (1989).
17. Kazovsky, L. G. Balanced phase-locked loops for optical homo-dyne receivers: performance analysis, hesign considerations, and laser linewidth requirements. *J. Lightwave Technol.* **LT-4**, 182-195 (1986).
18. Kikuchi, K., Impact of 1/f-type FM noise on coherent optical communications. *Electron. Lett.* **23**, 885–887 (1987).
19. Kazovsky, L. G., Performance Analysis and Laser Linewidth Requirements for Optical PSK Heterodyne Communications Systems. *J. Lightwave Technol.* **LT-4**, 415-425 (1986).
20. Fleming, M. W. & Mooradian, A. Spectral characteristics of external cavity controlled semiconductor lasers. *IEEE J. Quantum. Electron.* **17**, 44-59 (1981).
21. Yamamoto, Y., Mukai, T. & Saito, S. Quantum phase noise and linewidth of a semiconductor laser. *Electron. Lett.* **17**, 327-329 (1981).
22. Daino, B., Spano, P., Tamburrini, M. & Piazzolla, S. Phase noise and spectral line shape in semiconductor lasers. *IEEE J. Quantum. Electron*. **19**, 630-631 (1983).
23. Chen, M., Meng, Z., Wang, J. & Chen, W., Ultra-narrow linewidth measurement based on Voigt profile fitting. *Opt. Express*. **23**, 6803-6808 (2015).
24. Bruce, S. D., Higinbotham, J., Marshall, I. & Beswick, P. H., An analytical derivation of a popular approximation of the Voigt function for quantification of NMR spectra. *J. Magn. Reson*. **142**, 57-63 (2000).
